# Use of Medications and Lifestyles of Hypertensive Patients with High Risk of Cardiovascular Disease in Rural China

**DOI:** 10.1371/journal.pone.0124484

**Published:** 2015-05-01

**Authors:** Guanyang Zou, Zhitong Zhang, John Walley, Weiwei Gong, Yunxian Yu, Ruying Hu, Jia Yin, Min Yu, Xiaolin Wei

**Affiliations:** 1 COMDIS Health Services Delivery Research Program, China Program, University of Leeds, Shenzhen, China; 2 Nuffield Centre for International Health and Development, University of Leeds, Leeds, United Kingdom; 3 Zhejiang Centre for Disease Control and Prevention, Hangzhou, China; 4 Schoo of Public Health, Zhejiang University, Hangzhou, China; 5 School of Public Health and Primary Care, Chinese University of Hong Kong, Hong Kong, China; University of Sao Paulo Medical School, BRAZIL

## Abstract

**Background:**

Hypertension, with a global prevalence of 40%, is a risk factor for cardiovascular diseases (CVD). We conducted an exploratory study in Zhejiang China to understand the prevention of CVD among hypertensive patients with a 10 year CVD risk of 20% or higher. We assessed current practices in a rural ‘township hospital’ (a primary care facility), and compared them with international evidence-based practice.

**Methods:**

A questionnaire survey was conducted to examine the use of modern drugs (antihypertensive drugs, statins and aspirin) and traditional drugs, compliance to medications and lifestyle among 274 hypertensive patients aged 40-74, with a CVD risk of 20% or higher (using the Asian Equation).

**Results:**

The majority (72%) were diagnosed with hypertension at township hospitals. Only 15% of study participants used two anti-hypertensive drugs, 0.7% took statin and 2.9% aspirin. Only 2.9% combined two types of modern drugs, while 0.4% combined three types (antihypertensives, statins and aspirin). Herbal compounds, sometimes with internationally rarely recommended drugs such as Reserpine were taken by 44%. Analysis of drug adherence showed that 9.8% had discontinued their drug therapy by themselves. 16% had missed doses and these were on less anti-hypertensive drugs than those who did not (t=-5.217, P=0.003). Of all participants, 28% currently smoked, 39% drank regularly and only 21% exercised frequently. The average salt intake per day was 7.1 (±3.8) g, while the national recommended level is 6g.

**Conclusion:**

The study revealed outdated and inadequate treatment and health education for hypertensive patients, especially for those who have high risk scores for CVD. There is a need to review the community-based guidelines for hypertension management. Health providers and patients should make a transition from solely treating hypertension, towards prevention of CVD. Health system issues need addressing including improving rural health insurance cover and primary care doctors’ capacity to manage chronic disease patients.

## Background

Cardiovascular disease (CVD) is one of the leading causes of mortality and morbidity worldwide. CVD accounts for 38% of total mortality in China [1]. However, prevention and control of the CVD in China is challenging. The risk of suffering from CVD is governed in part by modifiable risk factors. Hypertension, with a global prevalence of 40%, is a consistent and independent risk factor for CVD. Hypertension contributes to approximately 55% of the global mortality caused by cardiovascular diseases. However, in most developing countries the health system fails to detect and manage hypertension effectively [2]. Studies showed that China’s urban areas had higher prevalence of hypertension than rural areas [2], but other studies found the prevalence was over 40% among rural residents in a northern province of China [3]. The national survey in 2002 showed that only 24% of hypertensive patients were aware of their condition, 19% were on treatment and the hypertension was only adequately controlled in only 4.5% of all the patients. Only 20% to 60% of patients took anti-hypertensive drugs [4]. A recent national study found that hypertension prevalence among people aged 45 years or older was nearly 40% and that over 40% of the hypertensive patients were unaware of their condition, with about half not receiving any medication and in 80% of all the patients the hypertension was not effectively controlled [1].

In China, the detection, treatment and control of hypertension has been improved since the implementation of the essential public health service and essential medicine system in China’s comprehensive reform in 2009 [2]. In Zhejiang province, the government dedicated RMB 40(1 USD = 6.2 RMB) per capita per year on rural township hospitals to deliver a defined package of public health services, including the management of chronic diseases, maternal and child care and annual health. The township hospitals, including the affiliated community health stations, are the major primary care providers in the rural areas. They are responsible for establishing the residents’ health records through annual free health examinations. These include a physical check, measure of blood pressure, and cardiograph examination for every resident in the catchment area of the township hospital. Diagnosis and consultation on anti-hypertensive treatment are provided whenever appropriate. An essential medicine list is operated in township hospitals with over 400 drugs, including modern anti-hypertensive drugs such as angiotensin converting enzyme inhibitors(ACEi), angiotensin receptor blockers(ARBs), thiazides, beta blockers and calcium channel blockers, as well as lipid regulating drugs (e.g., statins) and antiplatelet drugs(e.g., aspirin). The essential drug list also includes ‘herbal compound’ of traditional herbal, often mixed with modern drugs. The commonly prescribed ‘herbal compound’ include, for example, *Zhenju antihypertensive drugs (formulated by wild chrysanthemum flower extract powder*, *nacreous layer powder*, *clonidine hydrochloride*, *hydrochlorothiazide and rutin); compound anti-hypertensive tablets (formulated by reserpine and other modern drugs)*. A general practitioner in the township hospital can prescribe ‘herbal compound’ antihypertensive drugs as needed. The traditional Chinese medicine, which provided 20% of the outpatient care [5] was popular among Chinese hypertensive patients. These drugs from the essential drug lists are often prescribed to patients with zero mark-up to the purchasing price. Almost all rural residents are covered by health insurance, called New Cooperative Medical Scheme (NCMS). NCMS aims to address catastrophic health expenditure due to inpatient services, but covers only minimal outpatient costs [2].

In China, hypertension is classified into three levels: Grade I (Systolic BP 140–159), II (Systolic BP160-179) and III (Systolic BP 180+) based on patient’s blood pressure levels [6,7]. Anti-hypertensive management is provided based on the CVD risk levels which consider both grade of hypertension and other risk factors such as smoking and organ damage. The categories are super high risk, high risk, medium risk and low risk. All hypertensive patients require life style education, regardless of risk levels. Hypertension of the high-risk or super high risk categories require immediate drug therapy while hypertension of lower risk categories requires further observation before drug therapy can be given. Different categories of antihypertensive drugs including herbal compound are recommended [6,7], although statins and aspirin are not recommended by the community-based guideline [7]. Lifestyle education includes improved diet, smoking cessation and increased exercise [6,7]. All hypertensive patients receive regular follow-up management from the township hospital doctors based on the risk categories, including clinical consultation and/or lifestyle education. No referral system is operated in China, so that hypertensive patients may bypass primary care facilites to seek care in county level or other large hospitals.

Internationally, assessing risk of CVD is different from that in China described above. Public health intervention aims to address the patients’ risk of CVD, calculated based on global risk factors [8]. Hypertensive patients with a calculated 10-year CVD risk of over 20% are regarded as high risk and in need of therapeutic and lifestyle interventions. Studies have shown that effect of lifestyle education [9,10] and the modern drugs, i.e., antihypertensives [11], statins [12] and aspirin [13,14], especially their low-dose combination [15,16] was positive on CVD prevention in hypertension (and other patients) with CVD high risk. Most studies regarding hypertension in China focused on the prevalence, awareness, drugs and lifestyle interventions among general hypertension patients [2–4,17,18], but very few were on hypertensive patients with high risk of CVD. We recently conducted a cross-sectional study which found that 6% of rural residents aged between 40 and 75 had a 10-year CVD risk of 20% or higher, with only 35% of the high risk subjects reportedly using any of the modern drugs (e.g., antihypertensives, statins and aspirin) [19]. However, it had included all residents with higher risk of CVD, not specifically those with hypertension. In this paper, we aim to understand the prevention of CVD among hypertensive patients with a CVD risk of 20% or higher over 10 years, through assessing the current practices against the international evidence in a rural primary care setting in Zhejiang, China.

## Methods

### Ethics statement

This study was approved by the Ethical Review Board of the University of Leeds, UK and Zhejiang Provincial Center for Disease Control and Prevention. Written consent was sought from all the study participants.

### Setting

This study was part of an exploratory study to understand the feasibility of conducting a cluster randomized controlled trial to implement a systematic CVD risk reduction package which includes drug therapy, lifestyle education and adherence support in rural Zhejiang province, China [20,21]. Zhejiang, located on China’s Eastern coast, is a relatively rich province with a population of 54 million. CVD is the leading cause of death in Zhejiang, with the population-based mortality rate being 2.0%, similar to the national level [22]. The prevalence of hypertension was 21% (urban 22%; rural 20%), slightly lower than that of the national level (24%) [22].

This study was conducted in August 2012, in a township hospital which serves a population of 50,000 [20,21]. In the township hospital, hypertension management was conducted by general practice teams. Each team consisted of a general practitioner, a nurse and an auxiliary staff such as pharmacist, sometimes facilitated by a public health doctor. Each team was responsible for the delivery of hypertensive care in 1–2 ‘designated’ villages, with an average population of 1,500–2,000. The hypertensive patients were expected to meet their general practitioner regularly based on their CVD risk categories. However, the public health team often met their patients at homes or public places such as the old people’s center to fulfill the follow-up targets set by the essential public health service. The team is also responsible for conducting health checkups for those insured through the NCMS scheme and updating the residents’ health records. Under the NCMS system, patients could be reimbursed up to 30% of their medical cost each time they receive the outpatient care and up to RMB450 per year in the township hospital.

### Data collection

Adults aged 40–74 who held permanent residency and had existing resident’s health records were included (n = 10,964). We calculated their risk of having any CVD event in the next 10 years based on their gender, age, systolic BP, total cholesterol and smoking status by using Asian Equation, which was developed using six cohorts of 172,077 individuals who were followed-up for 20 years in several Asian countries [23]. Using this method we identified 721(6.6%) participants as having a 10-year CVD risk of 20% or higher. In total, 547(76%) were included after excluding those with serious illnesses such as later-stage cancer, while 393 (72%) provided informed consent to complete a questionnaire. The process of screening, including the exclusion criteria have been described elsewhere [20]. The questionnaire aimed to investigate the history of medications, lifestyles and their health seeking behavior. Primary care staff, who were generally familiar with hypertensive patients, were trained to conduct the survey in the township hospital. In this paper, we focus on the prevention for CVD among hypertensive patients. Therefore, we excluded 119 participants, including 3 participants with CVD only, 9 with diabetes only, 12 with both hypertension and CVD, 5 with CVD, hypertension and diabetes and 90 without any confirmed diseases. In total, we included 274 subjects (70%) who had hypertension, including those combined with diabetes and hyperlipidemia.

### Analysis

Data was analyzed using SPSS 18.0 (SPSS Inc, Chicago, USA). Descriptive analysis, Chi-square test were employed when appropriate. We measured the following practices of hypertensive management in the past 12 months.

Firstly we measured the proportion of patients taking at least one of the modern drugs(anti-hypertensives, statin and aspirin) or traditional drugs. Specifically, we measured the proportion of patients taking at least one of the above modern drugs, at least one anti-hypertensive drug, at least two anti-hypertensive drugs, aspirin and statin, especially combination of two or three types of the modern drugs (anti-hypertensives, aspirin and statin). We also measured the proportion of patients taking traditional drugs, such as ‘herbal compounds’ which sometimes include internationally rarely recommended drugs, such as Reserpine. In addition, we measured finanical burden of hypertensive treatment, i.e., average annual drug cost as proportion of general medication and annual per capita income.

Secondly, we measured compliance to any of the drugs including modern and traditional drugs. Specifically, we calculated the number of patients who had self-medicated, as a proportion of patients who took drugs; among the patients who took drugs because of a doctor’s advice, the proportion of patients who had missed doses, taken drugs incorrectly or irregularly, stopped taking drugs by themselves. Self-medication was defined as taking drugs without the doctor’s advice. If the patient had taken more doses than as advised by a doctor, it was defined as overdosing. If the patient had missed any doses as advised by a doctor, it was defined as a missing dose. If the patient had taken a different drug to that advised by the doctor, it was defined as taking drugs incorrectly. If the patient had taken the drugs at a time not suggested by a doctor, it was defined as taking drugs irregularly. Stopping taking drugs by themselves meant a patient stopping using drugs without the doctor’s advice.

Thirdly, we measured related lifestyles, including smoking (measured by the proportion of patients who reported to the doctor current smoking, daily smoking and daily consumption of cigarettes); and drinking (proportion of patients who drank alcohols, and drank on a daily basis); exercise (proportion of patients who exercised frequently, and frequency of exercise per patient per week, and proportion of patients who exercised>30 minutes each time); salt intake (average salt intake per day per family member).

Finally, we measured healthcare seeking behaviour of patients, in terms of number of visits to different types of healthcare facilities including primary care facilities (i.e., township hospitals and community health stations) and hospitals at or above county levels.

## Results

### General and clinical characteristics of the participants

Among the 274 hypertensive patients the average age was 69(±5.9), 77% were married and 92% had received education at primary school level only or were iliterate. Each subject had an average of 2.4 (±1.3) family members. The average annual income per capita was RMB 10,553(±26,866). On average, each subject had a history of hypertension of 7.6 (±6.5) years. Most (n = 188, 72%) of the subjects were diagnosed in the township hospitals. Each subject spent RMB1,297 (±2,046) on treatment of any diseases. The proportion spent on medical costs compared to their annual per capita income was 33% (±81.5). The average body mass index, systolic BP, diastolic BP, and total cholesterol was 25(±3.6), 165 (±15) mmHg, 85 (±13) mmHg, 4.1 (±1.2) mmol/L, respectively. The average 10-year risk of CVD was 33%(±11.1). Men had significantly higher marriage rate (P<0.001), more family members (P = 0.011), lower systolic BP (P<0.001) and lower 10-year risk of CVD (P = 0.033) than women (Table 1). The diastolic BP was positively related to the body mass index (Pearson r = 0.165, p = 0.018).

**Table 1:** General and clinical characteristics of hypertensive patients with high risk of CVD.

	Male	Female	Total
No. of participants, N (%)	155	119	274
Age, $\overset{-}{X}$±SD	68.4(±5.0)	68.9(±7.2)	68.8(±5.9)
Married, N (%)	135(87.1)^a^	77(64.7)	212(77.4)
Education			
Primary school and below, N (%)	137(88.4)	115(96.6)	252(92.0)
Junior high school, N (%)	14(9.0)	4(3.4)	18(6.6)
Senior high school and above, N (%)	4(2.6)	0	4(1.5)
No. of family members, $\overset{-}{X}$±SD	2.5(±1.3)^b^	2.3(±1.4)	2.4(±1.3)
1, N (%)	16(10.3)	35(29.4)	51(18.6)
2, N (%)	103(66.5)^c^	58(48.7)	161(58.8)
3 and more, N (%)	36(23.2)^d^	26(21.8)	62(22.6)
Average annual family income(RMB)**, $\overset{-}{X}$±SD	37815(±120984)	28835(±91411)	33932(±109086)
Annual per capital income (RMB), $\overset{-}{X}$±SD	11896(±31816)	9876(±22735)	10553(±26866)
History of hypertension(years), $\overset{-}{X}$±SD	7.1(±6.4)	8.2(±6.6)	7.6(±6.5)
Place of diagnosis			
National hospital, N (%)	2(1.4)	0	2(0.8)
Provincial/prefectural hospital, N (%)	5(3.4)	3(2.6)	8(3.0)
County hospital, N (%)	21(14.3)	14(12.1)	35(13.3)
Township hospital, N (%)	109(74.1)	79(68.1)	188(71.5)
Private clinic, N (%)	10(6.8)	20(17.2)	30(11.4)
Average medical cost per capita(2011) (RMB), $\overset{-}{X}$±SD	1305(±2110)	1286(±1971)	1297(±2046)
Average proportion of medical cost to annual per capital income**, (%) $\overset{-}{X}$±SD	32.6(±78.6)	33.0(±85.5)	32.8(±81.5)
Results of annual health check			
Body mass index, $\overset{-}{X}$±SD	24.7(±3.4)	24.6(±3.9	24.6(±3.6)
Systolic BP (mmHg), $\overset{-}{X}$±SD	161(±15)^e^	169(±14)	165(±15)
Diastolic BP (mmHg), $\overset{-}{X}$±SD	86(±13)	83(±11)	85(±13)
Total cholesterol (mmol/L), $\overset{-}{X}$±SD	4.0(±1.2)	4.2(±1.2)	4.1(±1.2)
Triglyceride(mmol/L), $\overset{-}{X}$±SD	1.2(±1.0)	1.5(±1.1)	1.3(±1.1)
Fasting Plasma Glucose (mmol/L), $\overset{-}{X}$±SD	5.4(±1.8)	5.8(±3.3)	5.6(±2.6)
10-year CVD risk(%), $\overset{-}{X}$±SD	31.9(±12.1)^f^	35.3(±9.5)	33.5(±11.1)

Significantly higher than female in

^a^(χ^2^ = 19.277,P<0.001),

^b^(Z = -2.556, P = 0.011),

^c^(χ^2^ = 16.719,P<0.001, compared with 1),

^d^(χ^2^ = 8.025, P = 0.005, compared with 1)

Significantly lower than female in

^e^(t = -3.872, P<0.001),

^f^(t = -2.147, P = 0.033)

*1 USD = 6.2 RMB

** The average of proportions of medical cost to annual per capital income of all participants.

### Service utilization

On average, each patient visited the township hospital 5.7 (±5.9) times. The average travel time from patient's home to township hospital was 14 (±7.8) minutes. Each patient visited the county hospital 0.6 (±1.4) times. The average time from patient's home to county hospital was 44 (±13.9) minutes. Each patient received 7 (±4.7) visits from the township hospital. Most (73%) of the patients wished to see doctors in the township hospitals, followed by hospitals in the county or above (15%), and community health stations or village clinics (9.5%)(Table 2).

**Table 2:** Health services utilization among hypertensive patients with high risk of CVD.

	Male	Female	Total
No. of participants, N (%)	155	119	274
Number of visits to township hospital in the last year	6.0(±6.1)	5.4(±5.7)	5.7(±5.9)
Average time from patient's home to township hospital(minutes)	13.8(±7.4)	15.2(±8.2)	14.4(±7.8)
Number of visits to county hospital in the last year	0.5(±1.0)	0.8(±1.8)	0.6(±1.4)
Average time from patient's home to county hospital(minutes)	44.0(±14.1)	45.0(±13.6)	44.4(±13.9)
Number of times that a patient was followed up by township hospital in the last year	6.8(±5.1)	7.2(±4.1)	7.0(±4.7)
Willingness to be treated in			
Hospital of county level or above	23(14.9)	19(16.0)	42(15.4)
Township hospital	114(74.0)	84(70.6)	198(72.5)
Village clinic	14(9.1)	12(10.1)	26(9.5)
Others	3(1.9)	4(3.4)	7(2.6)

### Use of medications

Among all the 274 participants, 89% took at least one of the modern drugs(anti-hypertensives, statin and aspirin) or traditional drugs; 63% took at least one of the above modern drugs; 63% took at least one of the anti-hypertensive drugs. Specifically, 48% took only one antihypertensive drug and 15% took at least two anti-hypertensive drugs. The most used anti-hypertensive drug was Calcium channel blocker (n = 126, 73%), followed by angiotensin coverting enzyme inhibitor (ACEi) or Angiotensin receptor blockers(ARBs) (n = 66, 38%), thiazide diuretic (n = 20, 12%), beta blockers (n = 6, 3.5%). Only 0.7% took statins, while 2.9% took aspirin. Only 2.9% combined two types of modern drugs(anti-hypertensives, statin and aspirin) and 0.4% combined three types as internationally recommended in hypertension (and other patients) with CVD high risk. However 44% took traditional drugs, such as ‘herbal compounds’, which sometimes include internationally rarely recommended drugs, such as Reserpine. On average, each patient spent RMB 36(±30.7) per month to purchase drugs for anti-hypertensive treatment (Table 3). The annual cost spent on these medication (RMB430) accounted for 33% of the general medical cost (RMB 1,297) and 4% of the average per capita annual income (RMB10,553).

**Table 3:** Treatment of hypertensive patients with high risk of CVD.

	Male	Female	Total
No. of participants, N (%)	155	119	274
Use of medications:			
At least one of the modern drugs (anti-hypertensives, statin, aspirin) or traditional drugs, N (%)	134(86.5)	109(91.6)	243(88.7)
At least one modern drug(anti-hypertensives, statin, aspirin)	101(65.2)	71(59.7)	172(62.8)
At least one anti-hypertensive drug, N (%)	101(65.2)	71(59.7)	172(62.8)
Beta blockers, N (%)	4(2.6)	2(1.7)	6(3.5)
ACEi* or ARBs, N (%)	40(25.8)	26(21.8)	66(38.4)
Thiazide diuretic, N (%)	9(5.8)	11(9.2)	20(11.6)
Calcium-Channel blocker, N (%)	72(46.5)	54(45.4)	126(73.3)
Only one anti-hypertensive drug, N (%)	80(51.6)	51(42.9)	131(47.8)
Two or more anti-hypertension drugs, N (%)	21(13.5)	20(16.8)	41(15.0)
Statins, N (%)	1(0.6)	1(0.8)	2(0.7)
Aspirin, N (%)	6(3.9)	2(1.7)	8(2.9)
Combing two types of modern drugs(anti-hypertensives, statin, aspirin)**	5(3.2)	3(2.5)	8(2.9)
Combining three types of modern drugs (anti-hypertensives, statin, aspirin), N (%)	1(0.6)	0	1(0.4)
Traditional drugs (e.g., herbal compounds), N (%)	65(41.9)	56(47.1)	121(44.2)
Drug cost per month (RMB), $\overset{-}{X}$±SD	35.2(±30.1)	36.6(±31.6)	35.8(±30.7)

*ACEi: Angiotensin converting enzyme inhibitor; ARB: angiotensin receptor blocker

** ‘Types’ of drugs refer to drugs for anti-hypertension, lipid regulation and antiplatelet.

### Medication compliance

Among 243 participants who took at least one of the modern drugs (anti-hypertensives, statin and aspirin) or traditional drugs, 3.8% reported they took drugs without receiving doctors’ advice. Of those who had taken drugs with doctors’ advice, 2.1% had overdosed, 16% had missed doses, 1.7% had taken incorrect drugs, and 19% had taken drugs irregularly, while 9.8% had discontinued the drugs by themselves (Table 4). On average, the participants who missed doses took significantly fewer drugs compared to those who did not miss doses (1.1 vs. 1.5, t = -5.217, P<0.001). The participants who stopped medication themselves also took significantly fewer drugs than those who did not (1.1 vs. 1.5, t = -4.689, P<0.001).

**Table 4:** Medication compliance of hypertensive patients with high risk of CVD.

	Male	Female	Total
Taking at least one CVD related drug, N (%)	134	109	243
Self-medicating (Taking drugs without doctor’s advice), N (%)	6(4.5)	3(2.8)	9(3.8)
Non self-medicating (Taking drugs with doctor’s advice), N (%)	128(95.5)	106(97.2)	234(96.3)
Overdosing, N (%)	4(3.1)	1(0.9)	5(2.1)
Missing drugs, N (%)	20(15.8)	18(17.0)	38(16.2)
Taking wrong drugs, N (%)	3(2.3)	1(0.9)	4(1.7)
Taking drugs irregularly, N (%)	25(19.5)	19(17.9)	44(18.8)
Stopping drugs by their own decision, N (%)	12(9.4)	11(10.4)	23(9.8)

### Lifestyles

Of all the 274 hypertensive patients, 76(28%) currently smoked, with an average consumption of 17 cigarettes (±10.1) per day. Among them, 87% reportedly smoked every day. Men had significantly higher smoking rate than women (46% vs. 4.2%, χ2 = 58.135, P<0.001). Among those who smoked, men consumed significantly more cigarettes each day than women (18 vs. 7, P = 0.009). Among those who did not currently smoke (n = 198), 88% had never smoked. Among the men, there were significantly less who had never smoked than women (74% vs. 98%, P<0.001). Of all, 106 (39%) of patients currently drank alcohol, 83% of whom reported drinking every day. More men reported drinking alcohol than women (57% vs 15%, P<0.001), or reported drinking on a daily basis than women (89% vs. 56%, P = 0.002). Among those who did not currently drink alcohol (n = 168), 88% never drank. On average, each subject exercised 5.5(±1.8) times per week, while 30% exercised>30 min each time, 21% reported exercising frequently. The average salt intake per day was 7.1 (±3.8)g per family member (Table 5).

**Table 5:** Lifestyles of hypertensive patients with high risk of CVD.

	Male	Female	Total
No. of participants, N (%)	155	119	274
Smoker, N (%)	71(45.8)^a^	5(4.2)	76(27.7)
Smoke occasionally, N (%)	8(11.3)	2(40.0)	10(13.2)
Smoke every day, N (%)	63(88.7)	3(60.0)	66(86.8)
Daily consumption of cigarettes, $\overset{-}{X}$±SD	17.5(±10.0)^b^	7.3(±6.2)	16.8(±10.1)
Non-smoker, N (%)	84(54.2)	114(95.8)	198(72.3)
Never smoke, N (%)	62(73.8)	112(98.2)	174(87.9)
Quit smoking for more than 1 year, N (%)	22(26.2)^c^	2(1.8)	24(12.1)
Alcohol drinker, N (%)	88(56.8)^d^	18(15.1)	106(38.7)
Drink occasionally, N (%)	10(11.4)	8(44.4)	18(17.0)
Drink every day, N (%)	78(88.6)^e^	10(55.6)	88(83.0)
Non-drinker, N (%)	67(43.2)	101(84.9)	168(61.3)
Never drink alcohol, N (%)	52(77.6)	96(95.0)	148(88.1)
Quit drinking for more than 1 year, N (%)	15(22.4)^f^	5(5.0)	20(11.9)
Doing physical exercises frequently, N (%)	31(20.3)	26(21.8)	57(21.0)
Times per week, $\overset{-}{X}$±SD	5.6(±2.0)	5.4(±1.6)	5.5(±1.8)
Minutes per time			
<15 min, N (%)	3(9.7)	2(8.0)	5(8.9)
15–30 min, N (%)	19(61.3)	15(60.0)	34(60.7)
>30 min, N (%)	9(29.0)	8(32.0)	17(30.4)
Daily per capital salt intake(g), $\overset{-}{X}$±SD	7.0(±3.8)	7.2(±3.8)	7.1(±3.8)
Weekly per capital pickle intake			
<250g	100(64.9)	73(61.9)	173(63.6)
250g-1000g	49(31.8)	43(36.4)	92(33.8)
>1000g	5(3.2)	2(1.7)	7(2.6)

Significantly higher than female in

^a^(χ^2^ = 58.135,P<0.001),

^c^(χ^2^ = 27.111,P<0.001),

^d^(χ^2^ = 49.227,P<0.001),

^e^(χ^2^ = 11.600,P = 0.002),

^f^(χ^2^ = 11.678,P = 0.001)

Significantly more than female in

^b^(t = 2.652, P = 0.009).

## Discussion

Our study reflects the current situation of prevention for hypertensive patients with a minimum risk of CVD in 10 years of 20% in the rural primary care setting. Our study finds that hypertensive patients tend to use township hospitals for diagnosis and usual care for hypertension. This may be because the primary care system is closer to patients’ homes and cheaper compared with big hospitals. Ideally, primary care facilities such as township hospitals should act as the first-contact to patients, and provide patient-centered, comprehensive and coordinated care for chronic patients [24].

Nearly 90% took any anti-hypertensive medication, while it was 50% in a national survey [2], 46.34% in a survey from Zhejiang [18] and 24.6% in a survey from minority group in Yunnan [17]. The better results of this study may be because our hypertensive participants were at higher risk of CVD so have better treatment awareness, while other studies included the general hypertensive patients. Over 60% took at least one of the modern drugs(antihypertensives, statins and aspirin), as compared to 47% in another similar study of assessing drug taking among high risk population which also included people without any known diseases [19]. Over 60% took at least one antihypertensive drug; however, only 15% took two anti-hypertensive drugs, suggesting the potential sub-optimal effect of hypertension control. Combing two anti-hypertensive drugs will not only strengthen the treatment effect, but will help to relive the adverse events of both drugs [11]. The pattern of using specific anti-hypertensive drugs was similar to another study where calcium channel blocker was most frequently used, followed by angiotensin converting enzyme inhibitors (ACEi), thiazide diuretics and beta blockers [19]. Similar to another similar study [19], we found less than 1% and 3% took statins and aspirin.

Effective prevention of CVD depends on simultaneously tackling of the multiple risks factors. Compared to international evidence-based recommendations, we found that only 2.9% combined two types of modern drugs (antihypertensives, statins and aspirin) and only 0.4% combined three types. There was a higher rate of using hypertensive drugs, but lower use of aspirin and statin. This reflects the reality of treating hypertension rather than comprehensive management of risk factors. China hypertension control guideline does mention the treatment of different risk factors for CVD prevention [6,7]. However, the community-based guideline does not include aspirin and statin, let alone the emphasis on the prescription of low-dose combination of modern drugs for the CVD prevention [7]. It also puts too high a threshold for drug recommendation as even hypertensive patients identified as ‘medium risk category’, where the risk of some cases may be over 20%, need to be observed before they are given drug therapy [6,7]. However, if more drugs such as statins are given, or the low-dose combination is taken, the actual medical cost will be higher than the medical cost as observed from this study. It may also cause higher financial burden for patients, as their current annual income spent on general medication was already up to 33%. The rural insurance cover on the outpatient care was rather limited to the extent that utilization of preventive care was restricted. On the patients’ side, the poor risk perception and care seeking of the hypertensive patients also need addressing to improve the uptake of modern CVD-related drugs for preventive purpose.

Nearly 50% had taken traditional drugs such as ‘herbal compound’ as compared to 23% in another study in China [19]. The drugs may include components which are rarely recommended by international guidelines such as Reserpine due to safety concerns. The popularity of these drugs could be due to the lower prices as compared to other modern medicine and traditional belief of patients [19]. However, the effects and safety of traditional drugs on the control of blood pressure and cholesterol levels especially the CVD events has not been well evaluated due to the lack of well-designed randomized controlled trials [25]. The national guidelines and essential drugs system may need reviewing, and in practice priority should always be given to prescribing and making affordable modern drugs for preventive purpose such as anti-hypertensive drugs and statins, which have been well evaluated on safety and effectiveness.

Most of patients took drugs because of the doctor’s advice. However, 16% of patients missed medication doses and 19% used drugs irregularly. Internationally the level of compliance with antihypertensive drug therapy was mixed [26,27], however comparison across studies was difficult due to the different measurement methods used. Our study is consistent with other studies that increase in the number of medications may increase compliance to anti-hypertensive drugs [27,28], though inconsistent with the finding of another study in Malaysia [26]. Probably due to the small sample size, we failed to detect significant relationships between medical compliance and other socio-economic indicators such as gender, education, marital status and cost of antihypertensive medications as identified by other studies [26,27]. To improve compliance to the therapeutic and lifestyle interventions, practical strategies could be explored such as the use of treatment supporters and text messages (SMS) [29].

China’s hypertension control guideline requires health education delivered to all hypertensive patients [6]. However, our results suggest the suboptimal effect of health education. Smoking rate among all the participants was 28%, higher than the national level(21%) [22]. Only 21% of patients exercised regularly, similar to the national level (24%) [22]. The average salt intake per family member was 7.1g, similar to the national average [22], but well above the WHO and nationally recommended level [6]. Changing the behavior of the high risk population is challenging in rural areas [30,31]. In China, health education is often conducted during primary care doctors’ routine follow-up visits, and often done in patients’ homes or public places such as an old people’s center. The quality of health education is thus difficult to be assured. Our study indicates the importance of systematic and individualized health education and consultation among hypertensive patients with high risk of CVD. This requires addressing of primary care doctors’ capacity with regards to their knowledge and skills to manage chronic disease patients [21].

This is an exploratory study which has helped to inform the design and conduct of a holistic intervention to reduce CVD event rates among diabetes and hypertensive patients with high risk of CVD in Zhejiang province, China [20,21]. Due to its exploratory nature, the generalisability of our results is limited as the study was conducted in only one township of China. In addition, the relatively small sample size has weakened the power to explore the impact of socio-economic and demographic factors on important variables such as BP levels, use of preventive drugs and medication adherence. Reporting bias is likely to exist in a quantitative survey despite enhanced training to the primary care staff to interview patients. Moreover, selection bias may exist as 28% of the participants with high risk of CVD were either unreachable or declined to participate in the survey. The relatively high loss to follow-up rate also indicates the difficulty of CVD preventive management. In this paper, our focus is not to understand to what extent the global risk based system will be overlapping with the risk classification system China is currently using, but to understand the medication, lifestyle and compliance of hypertensive patients with high risk of CVD. Further study should be conducted to compare the validity of these two systems to identify high risk hypertensive patients. However, it is important that CVD prevention should prioritize the management of hypertensive patients with high risk of CVD in high prevalence settings with limited resources.

## Conclusion

The study revealed outdated and inadequate treatment and health education for hypertensive patients, especially for those who have high risk score for CVD. There is a need to review the community-based guidelines for hypertension management. Health providers and patients should make a transition from just treating hypertension, towards prevention of CVD. Health system issues need addressing including improving rural health insurance cover and primary care doctors’ capacity to manage chronic disease patients.
